# Medication beliefs and adherence during and after pregnancy among women at risk for gestational hypertensive disorders

**DOI:** 10.3389/fdsfr.2025.1610273

**Published:** 2025-08-11

**Authors:** Pauline Dreesen, Dorien Lanssens, Sandy Nouwen, Pauline Volders, Febe Janssen, Adelheid Soubry, Wilfried Gyselaers, Michael Ceulemans

**Affiliations:** ^1^ Faculty of Medicine and Life Sciences, Limburg Clinical Research Center, Hasselt University, Diepenbeek, Belgium; ^2^ Future Health, Ziekenhuis Oost-Limburg, Genk, Belgium; ^3^ Department of Midwifery and Nursing Sciences, Center for Research and Innovation in Care, Antwerp University, Antwerp, Belgium; ^4^ Department of Human Genetics, Epigenetic Epidemiology Lab, Faculty of Medicine, KU Leuven, Leuven, Belgium; ^5^ Department of Obstetrics and Gynaecology, Ziekenhuis Oost-Limburg, Genk, Belgium; ^6^ Clinical Pharmacology and Pharmacotherapy, Department of Pharmaceutical and Pharmacological Sciences, KU Leuven, Leuven, Belgium; ^7^ IQ Health Science, Radboud University Medical Center, Nijmegen, Netherlands; ^8^ Child and Youth Institute, KU Leuven, Leuven, Belgium; ^9^ Research Foundation Flanders (FWO), Brussels, Belgium

**Keywords:** aspirin, gestational hypertension, preeclampsia, adherence, beliefs

## Abstract

**Introduction:**

Low-dose aspirin initiated ≤16 weeks of gestation reduces the risk of developing early-onset preeclampsia. However, no recent data are available on women’s beliefs about medication and adherence in pregnant women at risk for gestational hypertensive disorders (GHD). This study aimed to evaluate medication beliefs and adherence in this high-risk population, and to explore the relationship between adherence, self-reported aspirin intake, and pregnancy and neonatal outcomes.

**Methods:**

Pregnant women at risk for GHD followed up via remote blood pressure monitoring and who were prescribed aspirin 160 mg/day were included (ClinicalTrials.gov ID NCT04031430). Women’s beliefs about medication (in general and during pregnancy) and adherence were assessed using the Beliefs about Medicine Questionnaire (BMQ) and the Probabilistic Medication Adherence Scale (ProMAS) administered during pregnancy (at inclusion), at 10–21 days and 4–6 months postpartum. Aspirin intake was self-reported in the MediSafe app. We did not intervene throughout the study.

**Results:**

A total of 73 participants were recruited at a median gestational age of 14.1 weeks (IQR:13.2–15.6). The mean pregnancy BMQ scores for overuse and harm were 10.6 ± 2.9 and 8.8 ± 2.2 on a total score of 20, respectively. A total of 95% agreed to have a higher threshold for taking medicines during pregnancy and 50% disagreed with refraining from using medicines during pregnancy. Similar positive attitudes towards medicines were observed postpartum. The mean ProMAS score in pregnancy was 10.3 ± 3.9 out of 18. Similar rates were observed at 10–21 days postpartum, while a trend toward lower adherence was seen at 4–6 months postpartum (mean score 8.9 ± 4.5). The mean “minimal” and “maximal” self-reported aspirin intake was 82.5% and 98.6%, respectively. ProMAS scores were positively correlated with self-reported aspirin intake. Uncomplicated pregnancies showed higher ProMAS scores during pregnancy compared to complicated pregnancies.

**Discussion:**

Women at risk for GHD involved in a clinical trial showed positive beliefs about medication use in general and during pregnancy throughout the perinatal period and reported high aspirin adherence rates. However, more research is needed to provide real-world adherence estimates in pregnancy and to assess the predictive utility of the ProMAS instrument to forecast adherence throughout pregnancy and adverse pregnancy and neonatal outcomes.

**Clinical Trial Registration:**

identifier NCT04031430.

## 1 Introduction

Gestational hypertensive disorders (GHD) are amongst the most common pregnancy complications, affecting 5%–10% of pregnancies worldwide. Hypertension in pregnancy is defined as systolic blood pressure ≥140 mmHg and/or diastolic blood pressure ≥90 mmHg. GHD can be classified, according to the International Society for the Study of Hypertension in Pregnancy (ISSHP), into chronic or essential hypertension, gestational hypertension, and preeclampsia (PE). The latter can be further classified as early-onset PE or late-onset PE if diagnosed before or after 34 weeks of gestation, respectively. Additionally, preeclamptic patients may progress to hemolysis, elevated liver enzymes, and low platelet count (HELLP syndrome), a severe and life-threatening pregnancy pathology (Brown et al., 2018). A wide range of complications have been associated with GHD, varying in severity from mild hypertension to multisystem conditions with potentially life-threatening consequences for both mother and fetus (Hung et al., 2022; Naruse et al., 2021). Several risk factors have been identified that contribute to the development of GHD, such as a high pre-pregnancy body mass index (BMI>30 kg/m^2^), advanced maternal age (≥40 years), a multiplet pregnancy, a history of previous pregnancy complications, familial hypertension, smoking, and maternal comorbidities (e.g., chronic hypertension, pre-gestational diabetes mellitus, auto-immune diseases, and chronic kidney disease) (Brown et al., 2018; Sole et al., 2021; Lewandowska, 2021).

The risk of developing GHD can be managed by preventive measures such as tight monitoring of blood pressure (Lansse et al., 2020), maintaining prenatal physical activity (Skow et al., 2017; Dreesen et al., 2024), and the prophylactic use of low-dose aspirin (Asaflow ^©^) started at or before 16 weeks of gestation (Brown et al., 2018; Rolnik et al., 2017). The latter has been associated with significant reductions in the incidence of early-onset PE (Rolnik et al., 2017). The study of Wright et al. proved the importance of adherence to aspirin in the high-risk population. They showed that pregnant women with adherence of ≥90% benefited from aspirin nearly two times more than those with adherence of <90% (Wright et al., 2017). This information is of high importance because medication non-adherence to Asaflow in high-risk pregnancies has previously been estimated to be between 21.4%–46.3% in a 2016 cohort of 24 Dutch women (Abheiden et al., 2016). Furthermore, non-adherence rates to aspirin intake during pregnancy ranging from 46% to 94% have been reported when evaluated outside the controlled environment of clinical trials (Abheiden et al., 2016; van Montfort et al., 2020; Vinogradov et al., 2024). Vinogradov et al. evaluated barriers and facilitators of adherence to low-dose aspirin in pregnancy and identified several key factors, such as “Insufficient knowledge,” “Necessity-Concerns balance,” “Access to medicine,” and “Social influence” (Vinogradov et al., 2024). Pregnant women may have substantial information needs regarding the use and safety of medications in pregnancy (Ceulemans et al., 2022). At the same time, pregnant women may have unrealistic perceptions of the teratogenic risks of medicines, resulting in suboptimal treatment (Widnes and Schjøtt, 2017; Nordeng et al., 2010; Ceulem et al., 2019). It has been shown that women’s beliefs about medication during pregnancy have an impact on their medication adherence. Negative beliefs about medication use during pregnancy can lead to poor medication adherence, potentially leading to adverse maternal and neonatal health outcomes (Nordeng et al., 2010; Twigg et al., 2016). Therefore, focusing on medication adherence during pregnancy is of clinical importance (Ceulemans et al., 2019; Almuhareb et al., 2024). To our knowledge, no recent data are available regarding women’s beliefs about medication and adherence and the interplay between both in a high-risk pregnancy population. Therefore, the research objective was to evaluate medication beliefs and adherence of women at risk for developing GHD and to explore the relationship between medication adherence assessments and self-reported aspirin intake during pregnancy with pregnancy and neonatal outcomes.

## 2 Materials and methods

### 2.1 Study design

This prospective, interventional study was conducted at the Department of Gynecology and Obstetrics of Ziekenhuis Oost-Limburg (ZOL, Genk, Belgium) in collaboration with Hasselt University between March 2021 and May 2024 (ClinicalTrials.gov ID: NCT04031430 – CAPROM sub-study). One of the secondary research objectives was to evaluate beliefs about medication and adherence during pregnancy and in the postpartum period, and to explore the relationship between medication adherence and self-reported aspirin intake during pregnancy with pregnancy and neonatal outcomes.

### 2.2 Study participants

Pregnant women at risk for GHD who were followed up via remote blood pressure monitoring as part of the Pregnancy Remote Monitoring (PREMOM II) project (Lansse et al., 2020) at Ziekenhuis Oost-Limburg (ZOL, Genk, Belgium) between March 2021 and May 2023 were eligible for participation in this study.

#### 2.2.1 Ethics approval statement

Approval by the leading and local Ethical Committees was obtained prior to the study onset (BE300201938651). Written informed consent was obtained from all participants before inclusion. The study was performed in accordance with the Declaration of Helsinki.

### 2.3 Medication registration

Low-dose aspirin (Asaflow^©^) 160 mg once daily was prescribed to all subjects and was initiated before 16 weeks of gestation and continued until 36 weeks of gestation, consistent with the Aspirin for Evidence-based Pre-eclampsia Prevention Trial (Lansse et al., 2020; Rolnik et al., 2017).

All participants were asked to install the application Medisafe^©^ (MediSafe Inc.) on their smartphones to register their daily medication use during pregnancy. Medisafe^©^ is a free medication schedule and reminder app compliant with the General Data Protection Regulation and ISO 27001:2013 certified and is available in Dutch. The applicability and feasibility of Medisafe^©^ for medication management have already been demonstrated in the literature (Morawski et al., 2017; Huang et al., 2019) but were not previously used in the pregnant population.

In the Medisafe^©^ app, medication intake could be registered as “taken,” “missed,” or “skipped.” If the medication was “taken” or “skipped,” subjects had to actively press the corresponding button in the app. Medication reminders could be snoozed for 10 min with a maximum rappel of 4 reminders per medication, after which the medication intake was automatically registered as “missed.” The research team only accessed the Medisafe data at the end of the study and thus did not interfere in case of non-adherence during the study period.

### 2.4 Questionnaires

Women’s overall medication beliefs and adherence were measured using two standardized questionnaires, with approval from the original authors. First, the beliefs about medication use in general and during pregnancy were assessed using the “Beliefs about Medicine Questionnaire” (BMQ) (Nordeng et al., 2010; Ceulem et al., 2019; Horne et al., 1999). Second, women’s medication adherence was assessed using the Probabilistic Medication Adherence Scale (ProMAS) (Kleppe et al., 2015). All study participants were asked to complete the Dutch-language version of the BMQ and ProMAS at three time points: during pregnancy (at inclusion), between day 10 and day 21 postpartum, and between 4 and 6 months postpartum. The study participants received a personal link via email to complete the questionnaires online (via Castor EDC) in their home setting at the corresponding measurement time. Reminders were sent after approximately 2 weeks of non-completion, with a maximum of 4 reminders per time point.

### 2.5 Maternal demographics and clinical characteristics

The following maternal demographics and clinical characteristics were recorded at inclusion: age, (pre-gestational) weight and BMI, race, smoking status, family history of cardiovascular and gestational hypertensive disorders, medical history of relevant co-morbidities (such as chronic hypertension, thrombophilia, kidney disease, diabetes mellitus type II, thyroid problem), medication use during pregnancy (at and after inclusion), own birth weight, information regarding the current pregnancy (estimated date of delivery, single or multiple pregnancy, mode of conception, gravidity, parity, spontaneous or induced abortion), and prior pregnancy complications among multipara.

### 2.6 Pregnancy and neonatal outcomes

The following pregnancy and neonatal outcomes were extracted after delivery from the electronic patient files: date of delivery, gestational age at delivery, mode of delivery, diagnosis of GHD according to the ISSHP guidelines (Brown et al., 2018), neonatal birth weight and percentile, sex, admission to the neonatal intensive care unit (NICU), and neonatal mortality. Birth percentiles were calculated based on the Belgian curves published by the Studiecentrum voor Perinatale Epidemiologie (SPE, Brussel, 2009).

### 2.7 Data analyses

The statistical analysis was performed with IBM SPSS^®^ Statistics. The study population, BMQ results, ProMAS results, and self-reported aspirin intake were analyzed using descriptive statistics. Continuous data are presented as mean ± standard deviation (SD) or median (interquartile range), depending on the normality of the data. Normality was checked via the Shapiro-Wilk test. Categorical data are presented as numbers (%). Comparison of the paired BMQ and ProMAS sum scores at the different time points (i.e., during pregnancy and in the postpartum period) were evaluated using the repeated measures ANOVA and the Wilcoxon signed rank Test for continuous and categorical (ordinal) data, respectively. Spearman’s Rho correlation analyses were performed between: the ProMAS adherence sum score assessed during pregnancy and in the early postpartum phase (10–21 days postpartum) and the self-reported aspirin intake; and the pregnancy BMQ sum score of the general statements (harm and overuse) and the self-reported aspirin intake. Pearson correlation analysis was performed between the BMQ sum score of the general statements (harm and overuse score) and the ProMAS adherence sum score assessed during pregnancy. The relationship between the ProMAS results assessed during pregnancy and in the early postpartum phase (10–21 days postpartum), the self-reported aspirin intake, and pregnancy and neonatal outcomes were explored descriptively and tested via one-sided Independent Samples T-test or the Mann-Whitney U test for continuous data, depending on the normality, and via the Chi-squared test for categorical (binary) data. A p-value <0.05 was considered statistically significant.

#### 2.7.1 Study population

Subjects were included in the analysis if they had reported at least one active registration of aspirin intake in the Medisafe app (“taken” or “skipped”), and/or if they had completed the general or pregnancy-specific BMQ statements and/or the ProMAS instrument during pregnancy.

#### 2.7.2 BMQ

The subject’s degree of agreement with the general statements (G1-G8) and the pregnancy-specific statements (P1-P9) was scored on a 5-point Likert scale (“totally agree,” “agree,” “uncertain,” “disagree,” “totally disagree”). A score for “overuse” was calculated as the sum of G1+G4+G7+G8. Similarly, a score for “harm” was calculated as the sum of G2+G3+G5+G6. In addition, all statements were trichotomized as follows: (totally) agree, (totally) disagree, and uncertain.

#### 2.7.3 ProMAS

Answer possibilities for the 18 ProMAS questions were: “yes, true” (coded with 1) or ‘no, not true’ (coded with 0). Items marked with (R) were reversed coded. The items were summed to provide an adherence score, indicating the degree to which the woman is adherent. Higher scores represent higher adherence rates. The adherence score was categorized into low (0–4), medium-low (5–9), medium-high (10–14), and high adherence (15–18). In addition, adherence scores were further dichotomized: (medium)-low adherent (0–9) and (medium)-high adherent (10–18).

#### 2.7.4 Self-reported aspirin intake

The self-reported aspirin intake during pregnancy was measured using the women’s registrations in their personal Medisafe app. The “minimal” percentage of aspirin intake was calculated as the number of entries reported as “taken” compared to the total number of entries (“taken” + “skipped” + “missed”). The “maximal” percentage of aspirin intake was calculated as the number of “taken” entries compared to the sum of “taken” + “skipped” entries, which equals the percentage of actual medication adherence considering the number of days aspirin intake (or not) was actively recorded by the study participant.

## 3 Results

### 3.1 Study population

**FIGURE 1:**
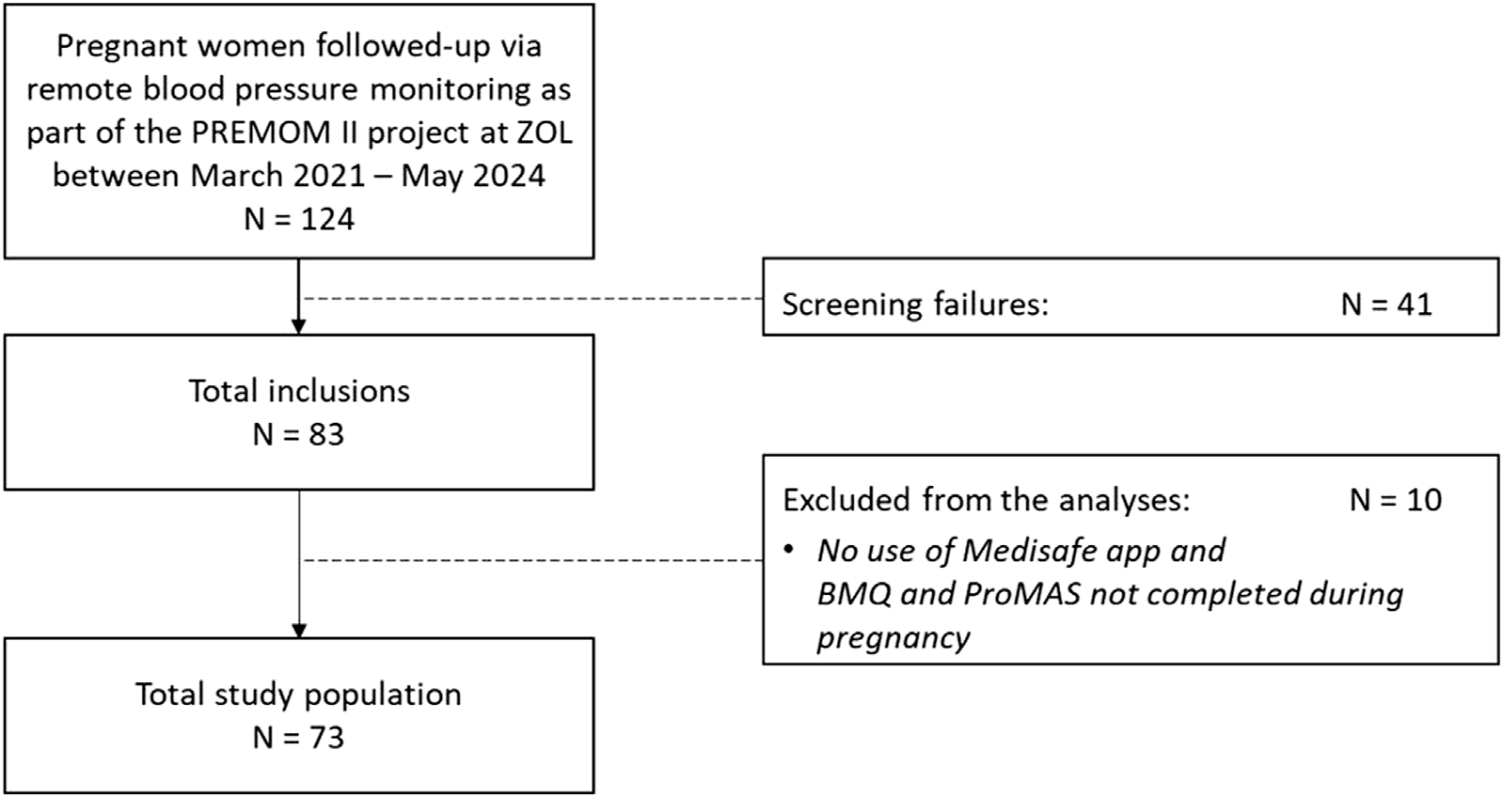
Flowchart of the total study population included in the statistical analyses. PREMOM II: Pregnancy Remote Monitoring II project; ZOL: Ziekenhuis Oost-Limburg; BMQ: Beliefs about Medicine Questionnaire; ProMAS: Probabilistic Medication Adherence Scale.

The study population involved 73 pregnant women at risk for GHD followed-up via prenatal remote blood pressure monitoring who completed the BMQ and/or ProMAS during pregnancy, and/or used the Medisafe app at least once for registration of their aspirin intake (Figure 1).

Detailed demographics and clinical characteristics of the total study population at inclusion are listed in Table 1. The median gestational age at inclusion was 14.1 weeks (IQR 13.2–15.6). A total of 84.9% of the women were nulliparous. All multipara (15.1%) had previous pregnancy complications. The mean maternal age was 30.1 ± 5.2 years, and the mean pre-gestational BMI was 29.1 ± 5.7 kg/m^2^, with the largest group (45.2%) being categorized with a BMI ≥30. At inclusion, 9.6% were still smokers in the ongoing pregnancy and 17 subjects (23.3%) reported pre-existing co-morbidities. A total of 21.9% used antihypertensive medication, and 9.6% reported no other medication intake apart from aspirin during the index pregnancy (Table 1).

**TABLE 1:** Demographics and clinical characteristics of the study population.

Total pregnancies	N = 73
Gestational age at inclusion, weeks	14.1 (13.2–15.6)
Maternal age, years	30.1 ± 5.2
≤19	1 (1.4%)
20–24	13 (17.8%)
25–29	15 (20.5%)
30–34	29 (39.7%)
35–39	13 (17.8%)
40–44	2 (2.7%)
Pre-gestational BMI, kg/m^2^	29.1 ± 5.7
<18.5	1 (1.4%)
18.5–24.9	20 (27.4%)
25.0–29.9	19 (26.0%)
≥30.0	33 (45.2%)
Smoking status	
Never	50 (68.5%)
Former	16 (21.9%)
Active	7 (9.6%)
Race	
White or Caucasian	66 (90.4%)
North African	6 (8.2%)
Middle Eastern	1 (1.4%)
Pre-existing co-morbidities	
Chronic hypertension	4 (5.5%)
Diabetes Mellitus I/II	3 (4.1%)
Thyroid problems	3 (4.1%)
Kidney disease	3 (4.1%)
Asthma	3 (4.1%)
Thrombophilia	1 (1.4%)
Family History of CV diseases	48 (65.8%)
Nulliparity	62 (84.9%)
Gravidity	
1	48 (65.7%)
2	18 (24.7%)
3	3 (4.1%)
>3	4 (5.5%)
History of spontaneous or induced abortion	
1	15 (20.5%)
2	2 (2.7%)
≥3	2 (2.7%)
Previous pregnancy complications	11 (15.1%)
Gestational hypertension	4 (5.5%)
Early-onset preeclampsia	3 (4.1%)
Late-onset preeclampsia	3 (4.1%)
HELLP	1 (1.4%)
Medication during pregnancy^a^	66 (90.4%)
Low-dose aspirin 160 mg	73 (100.0%)
Anti-hypertensive agents	16 (21.9%)
⁃ Labetalol	11 (15.1%)
⁃ α-methyl-L-DOPA	3 (4.1%)
⁃ Amlodipine	2 (2.7%)
⁃ Nifedipine	3 (4.1%)
⁃ Clonidine	1 (1.4%)
⁃ Isosorbide dinitrate	1 (1.4%)

Continuous data are given as mean ± standard deviation or median (interquartile range), depending on the normality of the data. Categorical data are presented as numbers (%). BMI: Body-mass index; CV: cardiovascular; HELLP: hemolysis elevated liver enzymes and low-platelets; IUGR: intra-uterine growth restriction.

^a^ Only medication that was taken during pregnancy and that is relevant to the research topic of GHD is shown.

Four women had a termination of their pregnancy because of intrauterine fetal death (n = 2), premature preterm rupture of membranes before 24 weeks of gestation (n = 1), or extreme fetal growth restriction (n = 1). Hence, pregnancy and neonatal outcomes are reported for 69 subjects (Table 2). The mean gestational age at delivery was 39.0 ± 1.5 weeks, with a mean birth weight of 3,212.1 ± 504.3 g. Four pregnancies (5.8%) ended prematurely (<37 weeks), and six (8.7%) small-for gestational age (SGA) neonates were born. The majority (65.2%) of the pregnancies were uncomplicated, although 27.5% and 4.3% developed GH or LPE, respectively. Almost 54% of the deliveries were induced and 11.6% of the neonates were admitted to the neonatal intensive care unit immediately after birth.

**TABLE 2:** Pregnancy and neonatal outcomes of the study population.

Total pregnancies	N = 69
Type of pregnancy	
Singleton	68 (98.6%)
Twin	1 (1.4%)
Mode of conception	
Natural	48 (69.6%)
Assisted conception	21 (30.4%)
Gestational age at delivery, weeks	39.0 ± 1.5
≥37 weeks	65 (94.2%)
<37 weeks	4 (5.8%)
Pregnancy complications related to GHD	
None	45 (65.2%)
Essential hypertension	2 (2.9%)
Gestational hypertension	19 (27.5%)
Late-onset preeclampsia	3 (4.3%)
Mode of delivery	
Vaginal birth	47 (68.1%)
Cesarean section	22 (31.9%)
Induced delivery	37 (53.6%)
Birth weight, g	3,212.1 ± 504.3
Birth weight ≤10th percentile	6 (8.7%)
Admission to NICU	8 (11.6%)

Continuous data are given as mean ± standard deviation. Categorical data are presented as numbers (%). NICU: neonatal intensive care unit.

### 3.2 BMQ and ProMAS

A total of 70 subjects completed the BMQ (general and/or pregnancy-specific statements) and/or the ProMAS during pregnancy at a mean gestational age of 17.0 (±4.6) weeks (min 12.4 - max 32.7 weeks). Of these, 50/70 (71.4%) completed both questionnaires during pregnancy. At the time of completion of the BMQ and/or ProMAS, 82.9% (58/70) of the women were already taking aspirin. The remaining group of women received the prescription for aspirin on the same day of completion of the questionnaire(s). Furthermore, 77.1% (54/70) of the women were taking other concomitant medication and/or supplements apart from aspirin at the time of completion of the questionnaire(s) (Supplementary Table S1).

After delivery, 48/70 (68.6%) and 39/70 (55.7%) subjects completed the BMQ and/or ProMAS at an average of 24.2 (±16.8) days and 5.89 (±1.1) months postpartum, respectively. Women completed the questionnaires at either one or both times postpartum. Thirteen women (18.6%) completed both the BMQ and ProMAS at all three time points.

No statistically significant correlation was found between the BMQ general statements (harm and overuse, p = 0.499 and p = 0.649, respectively) and the ProMAS adherence scores assessed during pregnancy (N = 56).

#### 3.2.1 Beliefs about medication use in general

The general statements of the BMQ were scored by 63 women during pregnancy and showed positive perceptions of medication use in general (Table 3). The mean scores for overuse and harm were 10.6 ± 2.9 and 8.8 ± 2.2 on a total score of 20, respectively. Similar positive attitudes towards medicines in general were observed at 10–21 days postpartum (Supplementary Table S2) and at 4–6 months postpartum (Supplementary Table S3), although there was a trend towards more “uncertain” agreements. The mean scores for overuse and harm of the women who completed the BMQ general statements postpartum are presented in Supplementary Table S4.

**TABLE 3:** Beliefs about medicines–general statements reported during pregnancy (N = 63).

BMQ general statements	(Totally) agree	Uncertain	(Totally) disagree
G1. Doctors use too many medicines.	11 (17.5%)	22 (34.9%)	30 (47.6%)
G2. People who take medicines should stop their treatment for a while every now and again.	12 (19.0%)	23 (36.5%)	28 (44.4%)
G3. Most medicines are addictive.	12 (19.0%)	11 (17.5%)	40 (63.5%)
G4. Natural remedies are safer than medicines.	15 (23.8%)	14 (22.2%)	34 (54.0%)
G5. Medicines do more harm than good.	2 (3.2%)	12 (19.0%)	49 (77.8%)
G6. All medicines are poisons.	2 (3.2%)	2 (3.2%)	59 (93.7%)
G7. Doctors place too much trust on medicines.	12 (19.0%)	16 (25.4%)	35 (55.6%)
G8. If doctors had more time with patients they would prescribe fewer medicines.	16 (25.4%)	16 (25.4%)	31 (49.2%)

**FIGURE 2:**
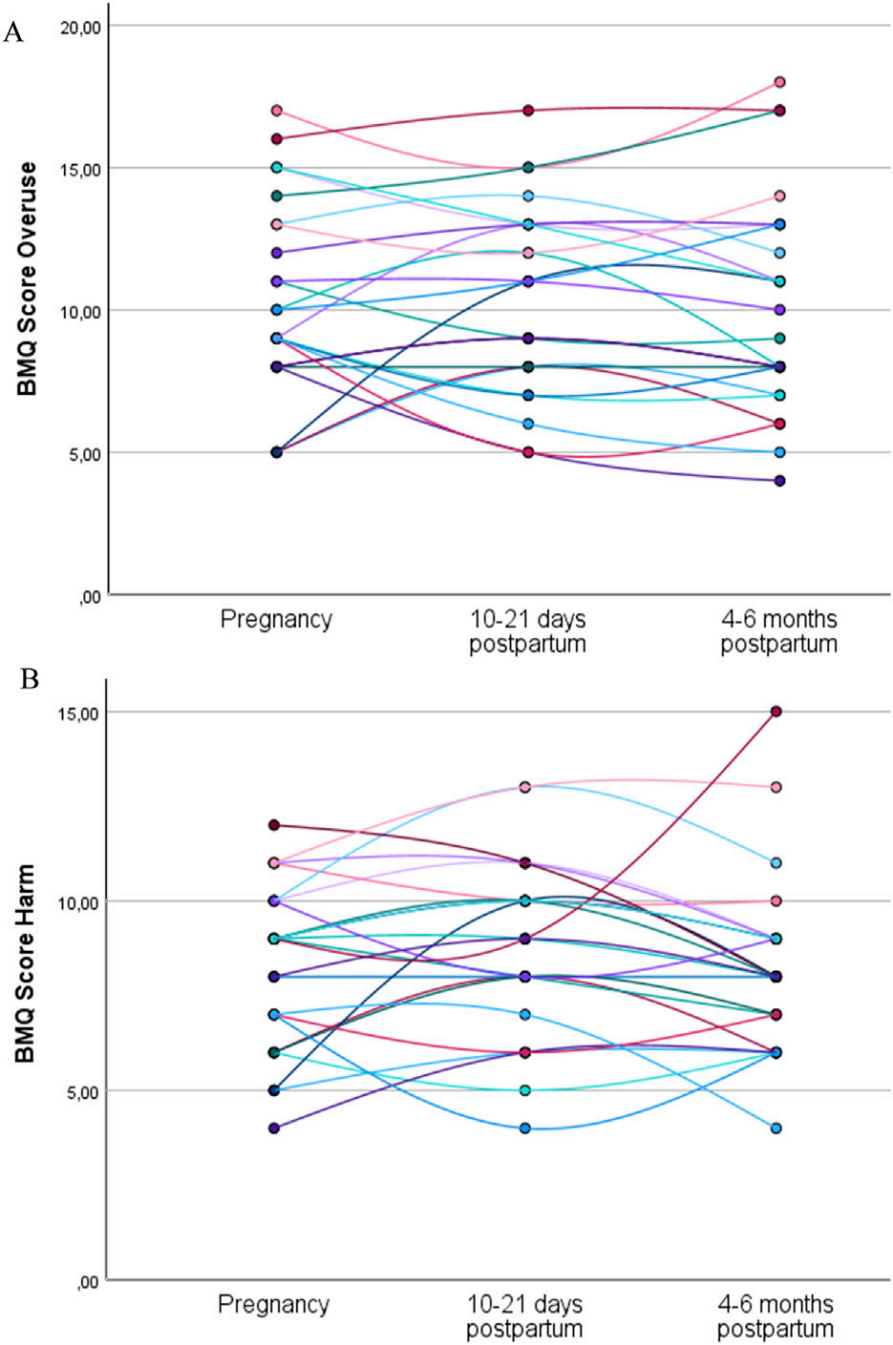
Individual trajectories of the overuse **(A)** and harm **(B)** scores for the women who completed the general statements of the BMQ during pregnancy, at 10–21 days postpartum, and at 4–6 months postpartum (N = 24). Each line represents the trajectory of an individual participant.

A total of 24 women (38%) completed the general statements of the BMQ during pregnancy, at 10–21 days postpartum and again at 4–6 months postpartum. The individual trajectories for the overuse and harm scores show variance in intrapersonal changes between the subjects throughout pregnancy and in the postpartum period (Figure 2).

Among the women who completed the BMQ during pregnancy and at both postpartum time points, the mean overuse and harm scores were not statistically significantly different between the three time points (Table 4).

**TABLE 4:** Overuse and harm scores of the women who completed the BMQ general statements during pregnancy and postpartum (N = 24).

BMQ score	During pregnancy (N = 24)	10–21 days postpartum (N = 24)	4–6 months postpartum (N = 24)	P-value
BMQ general - score overuse (/20)	10.4 ± 3.4	10.5 ± 3.3	10.2 ± 3.9	0.804
BMQ general - score harm (/20)	8.3 ± 2.2	8.7 ± 2.3	8.2 ± 2.4	0.305

Continuous data are given as mean ± standard deviation. BMQ: Beliefs about Medicine Questionnaire. P-value was assessed via repeated measures ANOVA. A p-value <0.05 was considered statistically significant.

#### 3.2.2 Beliefs about medication use during pregnancy

A total of 60 women completed the pregnancy-specific statements of the BMQ at 17.0 (±4.6) weeks of gestation (Table 5). Half of the women disagreed with refraining from using medicines during pregnancy (P2), although 95% agreed to have a higher threshold for taking medicines during pregnancy (P3). The majority of the women acknowledged the potential benefits of medication use during pregnancy (P1, P4, P5, P6). The preferences towards the use of natural remedies during pregnancy are somewhat positive to uncertain (P7, P8), although 66.7% agreed to consult a doctor first before the use of natural remedies during pregnancy (P9).

**TABLE 5:** Beliefs about medicines–pregnancy-specific statements reported during pregnancy (N = 60).

BMQ pregnancy-specific statements	(Totally) agree	Uncertain	(Totally) disagree
P1. All medicines can be harmful for the fetus.	9 (15.0%)	12 (20.0%)	39 (65.0%)
P2. Even if I am ill and if not pregnant would have taken a medicine, I believe it’s better for the fetus that I refrain from using medicines during pregnancy.	22 (36.7%)	8 (13.3%)	30 (50.0%)
P3. I have a higher threshold for using medicines when I am pregnant than when I am not pregnant.	57 (95.0%)	1 (1.7%)	2 (3.3%)
P4. Thanks to treatment with medicines during pregnancy, lives of many unborn children are saved each year.	29 (48.3%)	31 (51.7%)	0 (0.0%)
P5. It is better for the fetus that I use medicines and get well than to have an untreated illness during pregnancy.	38 (63.3%)	18 (30.0%)	4 (6.7%)
P6. Doctors prescribe too many medicines to pregnant women.	2 (3.3%)	20 (33.3%)	38 (63.3%)
P7. Natural remedies can generally be used by pregnant women.	21 (35.0%)	28 (46.7%)	11 (18.3%)
P8. Pregnant women should preferably use natural remedies during pregnancy.	21 (35.0%)	24 (40.0%)	15 (25.0%)
P9. Pregnant women should not use natural remedies without the consent of a doctor.	40 (66.7%)	16 (26.7%)	4 (6.7%)

Of the women who completed the pregnancy-specific statements of the BMQ at 10–21 days postpartum (Supplementary Table S5), a higher percentage agreed with refraining from using medicines during pregnancy (P2) (48.7% vs. 36.7%). In contrast, a higher percentage of women disagreed that it is better for the fetus to use medicines than to have an untreated illness during pregnancy (P5) (17.9% vs. 6.7%). At 4–6 months postpartum (Supplementary Table S6), again approximately 50% disagreed with refraining from using medicines during pregnancy (P2), although a higher percentage agreed that thanks to treatment with medicines during pregnancy the lives of many unborn children are saved every year (P4) (64.7% vs. 48.3%) compared to the pregnancy questionnaire.

#### 3.2.3 ProMAS

A total of 61 women completed the ProMAS during pregnancy and had a mean score of 10.3 ± 3.9 out of 18. If the individual adherence scores were categorized, 6.6% of the participants were considered non-adherent, 37.7% were medium-low adherent, 36.1% were medium-high adherent, and 19.7% were high adherent. Similar rates were observed in the group of women who completed the ProMAS at 10–21 days postpartum, while at 4–6 months postpartum, there was a trend towards a lower mean score with an increase in low adherence and a decrease in high adherence rates (Table 6).

**TABLE 6:** Results of the ProMAS completed during pregnancy and postpartum.

ProMAS score	During pregnancy (N = 61)	10–21 days postpartum (N = 38)	4–6 months postpartum (N = 34)
ProMAS – sum score	10.3 ± 3.9	9.8 ± 4.5	8.8 ± 4.4
Low adherence (0–4)	4 (6.6%)	5 (13.2%)	7 (20.6%)
Medium-low adherence (5–9)	23 (37.7%)	13 (34.2%)	11 (32.4%)
Medium-high adherence (10–14)	22 (36.1%)	14 (36.8%)	14 (41.2%)
High adherence (15–18)	12 (19.7%)	6 (15.8%)	2 (5.9%)

Continuous data are given as mean ± standard deviation or median (interquartile range), depending on the normality. Categorical data are presented as numbers (%). ProMAS: Probabilistic Medication Adherence Scale.

Response rates to the individual items of the ProMAS completed during pregnancy, at 10–21 days postpartum, and/or at 4–6 months postpartum, are shown in Table 7. At 4–6 months postpartum, a higher percentage of women stated that they sometimes take less medicines than prescribed by their doctor and that it has happened (at least once) that they changed the dose of (one of) their medicines without discussing this with their doctor compared to during pregnancy (41.2% vs. 14.8% and 32.4% vs. 16.4%, respectively).

**TABLE 7:** Probabilistic Medication Adherence Scale (ProMAS) reported during pregnancy and postpartum.

ProMAS statements	During pregnancy (N = 61)	10–21 days postpartum (N = 38)	4–6 months postpartum (N = 34)
1. It has happened at least once that I forgot to take (one of) my medicines. (R)	41 (67.2%)	24 (63.2%)	27 (79.4%)
2. It happens occasionally that I take (one of) my medicines at a later moment than usual. (R)	52 (85.2%)	33 (86.8%)	32 (94.1%)
3. I have never (temporarily) stopped taking (one of my) medicines.	23 (37.7%)	13 (34.2%)	9 (26.5%)
4. It has happened at least once that I did not take (one of) my medicines for a day. (R)	43 (70.5%)	22 (57.9%)	24 (70.6%)
5. I am positive that I have taken all the medication that I should have taken in the previous year.	22 (36.1%)	14 (36.8%)	12 (35.3%)
6. I take my medicines exactly at the same time every day.	9 (14.8%)	6 (15.8%)	5 (14.7%)
7. I have never changed my medicine use myself.	35 (57.4%)	18 (47.4%)	18 (52.9%)
8. In the past month, I forgot to take my medicine at least once. (R)	23 (37.7%)	15 (39.5%)	13 (38.2%)
9. I faithfully follow my doctor’s prescription concerning the moment of taking my medicines.	43 (70.5%)	27 (71.1%)	24 (70.6%)
10. I sometimes take (one of) my medicines at a different moment than prescribed (e.g., with breakfast or in the evening). (R)	11 (18.0%)	13 (34.2%)	12 (35.3%)
11. In the past, I once stopped taking (one of) my medicines completely. (R)	24 (39.3%)	17 (44.7%)	14 (41.2%)
12. When I am away from home, I occasionally do not take (one of) my medicines. (R)	28 (45.9%)	17 (44.7%)	19 (55.9%)
13. I sometimes take less medicine than prescribed by my doctor. (R)	9 (14.8%)	8 (21.1%)	14 (41.2%)
14. It has happened (at least once) that I changed the dose of (one of) my medicines without discussing this with my doctor. (R)	10 (16.4%)	5 (13.2%)	11 (32.4%)
15. It has happened (at least) once that I was too late with filling a prescription at the pharmacy. (R)	18 (29.5%)	13 (34.2%)	16 (47.1%)
16. I take my medicines every day.	52 (85.2%)	31 (81.6)	25 (73.5%)
17. It has happened (at least once) that I did not start taking a medicine that was prescribed by my doctor. (R)	25 (41.0%)	20 (52.6%)	18 (52.9%)
18. I sometimes take more medicines than prescribed by my doctor. (R)	3 (4.9%)	4 (10.5%)	2 (5.9%)

Twenty-two women completed the ProMAS at all three time points (Table 8). For this specific group, there was (also) a trend towards a lower mean sum score at 4–6 months postpartum compared to during pregnancy (8.9 ± 4.5 vs. 10.5 ± 3.8), although only borderline significant. In addition, there was a trend towards a higher percentage of participants categorized as low adherent and medium-low adherent, although not statistically significant.

**TABLE 8:** Sum scores and adherent rates of the women who completed the ProMAS both during pregnancy and early and late postpartum (N = 22).

ProMAS score	During pregnancy (N = 22)	10–21 days postpartum (N = 22)	4–6 months postpartum (N = 22)	P-value
ProMAS – sum score	10.5 ± 3.8	9.7 ± 4.5	8.9 ± 4.5	0.075
Low adherence (0–4)	2 (9.1%)	3 (13.6%)	4 (18.2%)	0.248
Medium-low adherence (5–9)	6 (27.3%)	8 (36.4%)	8 (36.4%)	
Medium-high adherence (10–14)	11 (50.0%)	8 (36.4%)	8 (36.4%)	
High adherence (15–18)	3 (13.6%)	3 (13.6%)	2 (9.1%)	

Continuous data are given as mean ± standard deviation or median (interquartile range), depending on the normality. Categorical data are presented as numbers (%). ProMAS: Probabilistic Medication Adherence Scale.

**FIGURE 3:**
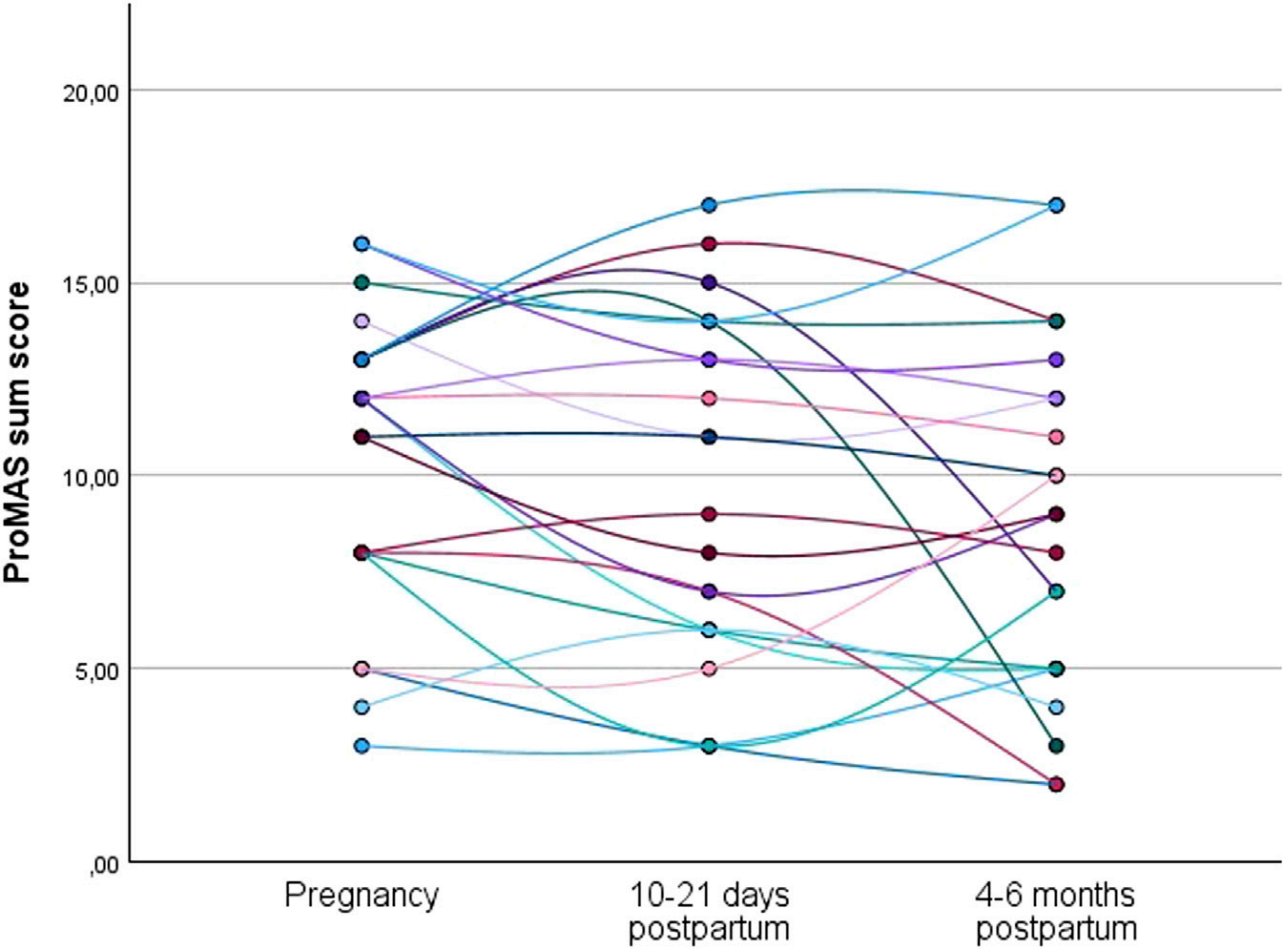
Individual trajectories of the medication adherence sum scores for the women who completed the ProMAS during pregnancy, at 10–21 days postpartum, and at 4–6 months postpartum (N = 22). Each line represents the trajectory of an individual participant.

Individual trajectories of the evolution in ProMAS sum scores during pregnancy, at 10–21 days postpartum, and at 4–6 months postpartum are shown in Figure 3. The individual trajectories for the medication adherence sum scores showed variance in intrapersonal changes between subjects throughout pregnancy and in the postpartum period.

### 3.3 Self-reported aspirin intake

A total of 67 (91.8%) subjects used the Medisafe app at least once to report their aspirin intake during pregnancy. The Medisafe app was used from 15.8 ± 4.3 weeks until 34.4 ± 3.8 weeks of gestation, with a mean duration of 18.6 ± 5.6 weeks. Thirteen (19.4%) subjects did not use the Medisafe app until the prescribed end date of aspirin intake (i.e., 36 weeks of gestation), of which four women had a pregnancy termination and one subject delivered preterm. The mean “minimal” and “maximal” self-reported aspirin intake was 82.5% (min 4.2%, max 100.0%) and 98.6% (min 79.4%, max 100%), respectively. A “minimal” aspirin intake level of ≥80% and ≥90% was reported in 74.6% (50/67) and 53.7% (36/67) of the women, respectively. With respect to the “maximal aspirin intake”, only one woman reported an intake level of <80% (i.e. 79.4%) and 97.0% (65/67) of the women had an intake level of ≥90%.

#### 3.3.1 Correlation between ProMAS/BMQ and self-reported aspirin intake

Of the Medisafe app users, 55 subjects also completed the ProMAS instrument during pregnancy. In this group, a total of 54.5% (30/55) and 98.2% (54/55) women had a “minimal” and “maximal” self-reported aspirin intake of ≥90%, respectively. Correlation analysis showed a positive correlation between the pregnancy ProMAS sum score and the “minimal” (*r* = 0.524, p < 0.001) (Supplementary Table S7) and “maximal” (r = 0.297, p = 0.028) self-reported aspirin intake (Supplementary Table S8); the higher the medication adherence ProMAS sum score, the higher the mean percentage of self-reported aspirin intake.

A total of 35 women who used the Medisafe app during pregnancy also completed the ProMAS at 10–21 days postpartum. The early postpartum ProMAS sum scores were also positively correlated with the “minimal” (r = 0.761, p < 0.001) (Supplementary Table S9) and “maximal” (r = 0.432, p = 0.010) self-reported aspirin intake during pregnancy (Supplementary Table S10).

No statistically significant correlation was found between the BMQ general statements (harm and overuse) assessed during pregnancy and the “minimal” (p = 0.992 and p = 0.692, respectively) and “maximal” (p = 0.424 and p = 0.517, respectively) self-reported aspirin intake during pregnancy (N = 57).

#### 3.3.2 Relationship between ProMAS, self-reported aspirin intake and outcomes

Table 9 shows an overview of the self-reported aspirin intake and the pregnancy ProMAS adherence rates for pregnancies with an uncomplicated outcome (N = 27) as well as for pregnancies complicated by GHD (N = 18), and/or preterm birth (N = 2), and/or an SGA neonate (N = 4). Most women with such a complicated pregnancy outcome (62.5%) reported medium-low ProMAS adherence rates during pregnancy, while women with an uncomplicated term pregnancy showed a higher percentage of medium-high adherence (63.0%). This was also reflected in the higher mean ProMAS sum scores reported during pregnancy in the group of uncomplicated pregnancies (10.9 ± 3.5) compared to the group of women with a complicated pregnancy (8.9 ± 4.1), although not statistically significant (p = 0.065, Supplementary Table S11). The “minimal” and “maximal” self-reported aspirin intake levels were similar between complicated and uncomplicated pregnancies.

**TABLE 9:** Overview of the ProMAS sum scores during pregnancy and the self-reported aspirin intake according to the pregnancy outcome (N = 51).

Pregnancy outcome	ProMAS pregnancy adherence rate		Minimal self-reported aspirin intake		Maximal self-reported aspirin intake	
	Medium-low (0–9)	Medium-high (10–18)	≥80%	≥90%	≥80%	≥90%
Uncomplicated pregnancy (N = 27)	10 (37.0%)	17 (63.0%)	20 (74.1%)	14 (51.9%)	27 (100.0%)	27 (100.0%)
Pregnancy complicated by GHD and/or PTB and/or SGA (N = 24)^a^	15 (62.5%)	9 (37.5%)	19 (79.2%)	14 (58.3%)	23 (95.8%)	23 (95.8%)
*GHD (N = 18)*	10 (55.6%)	8 (44.4%)	16 (88.9%)	13 (72.2%)	18 (100.0%)	18 (100.0%)
*PTB (N = 2)*	2 (100.0%)	0 (0.00%)	1 (50.0%)	0 (0.00%)	1 (50.0%)	1 (50.0%)
*SGA (N = 4)*	3 (75.0%)	1 (25.0%)	2 (50.0%)	1 (25.0%)	4 (100.0%)	4 (100.0%)

ProMAS: Probabilistic Medication Adherence Scale; GHD: gestational hypertensive disorders; PTB: preterm birth; SGA: small for gestational age neonate.

^a^ In case the subject was diagnosed with any subtype of GHD in combination with PTB, and/or SGA, the subject was categorized under “GHD.” This was the case for four subjects: two women also delivered preterm, and two other women also had an SGA neonate. None of the subjects classified under “SGA” or “PTB” had any other complication.

The ProMAS adherence rates assessed in the early-postpartum phase (i.e., at 10–21 days postpartum) showed similar results between uncomplicated and complicated pregnancies (Supplementary Table S12), while the “minimal” and “maximal” self-reported aspirin intake levels were higher for women with an uncomplicated pregnancy outcome, although not statistically significantly different (Supplementary Table S13).

## 4 Discussion

The main findings of this study are that pregnant women at risk for GHD have positive beliefs about medication use in general and during pregnancy and show high levels of self-reported aspirin intake and high medication adherence rates during pregnancy.

### 4.1 Beliefs about medication use in general and during pregnancy

This study showed that pregnant women at risk for GHD have positive beliefs about medication use in general, which is in line with other studies reporting positive attitudes toward medicines in general in the pregnant population (Nordeng et al., 2010; Ceulem et al., 2019; Zaki and Albarraq, 2014; Tefera et al., 2020). The positive perceptions of medication use observed in our at-risk study population also remained in the postpartum period. The mean scores for overuse and harm showed no statistically significant difference between pregnancy, at 10–21 days postpartum, and at 4–6 months postpartum. Based on these results, at the population level, the pregnancy or postpartum status may not change women’s overall perception of medication use. However, individual changes throughout the perinatal period cannot be ruled out and require clinical attention.

While 95% of the women at risk for GHD agreed to have a higher threshold for taking medicines during pregnancy, only 37% agreed to refrain from using medicines during pregnancy. Higher thresholds for taking medicines in pregnancy have previously been reported multiple times (Nordeng et al., 2010; Ceulem et al., 2019; Zaki and Albarraq, 2014). However, in the “general” pregnant population, including both low- and high-risk pregnancies, more women (58%–78.9%) agreed that it is better for the fetus to refrain from using medicines during pregnancy (Nordeng et al., 2010; Ceulem et al., 2019; Zaki and Albarraq, 2014; Tefera et al., 2020). Despite a limited sample size and the high percentage of nulliparous women, our study showed that an at-risk pregnant population enrolled in a clinical trial context may be more aware of the potentially beneficial effects of medicines used during pregnancy compared to the general pregnant population. This could be the result of our study population having been well-informed about their risk of developing GHD and the importance and effectiveness of prophylactic aspirin intake in pregnancy. The importance of being well-informed by a healthcare professional on medication adherence during pregnancy has been reported in the literature (Vinogradov et al., 2024; Nordeng et al., 2010; Ceulem et al., 2019; Mortelmans et al., 2024). However, similar perceptions regarding the use of natural remedies during pregnancy were found between our at-risk and the general pregnancy population and require careful consideration from clinicians as risks of herbal products in pregnancy cannot be ruled out (Ceulem et al., 2019). In the postpartum period, intra- and interindividual differences were noted for certain statements compared to during pregnancy. This highlights again that clinicians, when providing counseling, should be well aware of the likelihood of differences and potential changes in women’s perceptions of medication during and after pregnancy (Bjertrup et al., 2025).

### 4.2 Medication adherence and self-reported aspirin intake

In our study cohort, 55.8% reported (medium)-high medication adherence rates during pregnancy, with a mean score of 10.3 ± 3.9 out of 18. Similar adherent rates were observed at 10–21 days postpartum but were lower at 4–6 months postpartum compared to pregnancy levels. This decline may be explained by the importance that these at-risk women attribute to medication use in pregnancy, with perceived benefits not only for themselves but also for their unborn child, in contrast to the (late) postpartum period when GHD and associated risks for the infant are no longer applicable. Another possibility is that over time, pregnancy experiences gradually fade away while women are regaining pre-pregnancy feelings and perceptions (Bjertrup et al., 2025). Furthermore, most women continue to take “pregnancy-related” medication early postpartum (e.g., antihypertensive medication, anticoagulants, etc.), which is no longer needed at 4–6 months postpartum (Brown et al., 2018). The lack of a significant result for the decrease in ProMAS sum scores during and after pregnancy could be due to the relatively small sample size in our study.

At the individual level, some women reported higher medication adherence sum scores late compared to early postpartum. Despite the wide variation in PROMAS scores among individual participants, a very high to maximum level of self-reported aspirin adherence in pregnancy was observed in this cohort. The mean “minimal” self-reported aspirin intake was 82.5%, while three-fourths of the pregnant women reported an intake level of ≥80%. Importantly, the calculation of the minimum self-reported aspirin intake clearly entails limitations (i.e., “missed” registrations could mean that aspirin was taken but not recorded in the app), potentially underestimating the actual adherence. In contrast, the maximum self-reported intake may provide a more reliable estimation of the actual adherence, approaching 100% for nearly all women. The high adherence rate observed in our cohort is likely an overestimation of the situation in the real world, caused by the inclusion of women willing to participate in a trial and to communicate about their medication adherence while being aware that their adherence was monitored. The use of the app, with its automatic notifications timed for aspirin and medication intake, may also have improved the adherence or at least minimized the likelihood of forgetting aspirin intake. Other cohorts in a clinical trial setting on aspirin compliance also reported high adherence levels (Rolnik et al., 2017; van Montfort et al., 2020).

Finally, a positive correlation was found between ProMAS scores, both during pregnancy and in early postpartum, and the self-reported aspirin intake during pregnancy. Although further research is needed, this finding paves the way for the potential clinical use of the ProMAS instrument in early pregnancy as a predictive tool for medication adherence in the (at-risk) pregnant population. Equally important, a trend toward higher ProMAS scores was noted among women with uncomplicated pregnancies compared to women experiencing complications such as GHD, preterm birth, or SGA. This intriguing observation also points to the potential value of assessing women’s medication adherence early in pregnancy with the ProMAS instrument, as it may help identify pregnant women at risk for adverse pregnancy and neonatal outcomes. This will enable timely clinical and behavioral interventions to reverse the course.

### 4.3 Strengths and limitations of the study

To the best of our knowledge, this was the first study assessing beliefs about medication and adherence among women at risk for developing GHD. Medication adherence was measured using both a standardized, generic instrument (ProMAS) as well as self-reported registrations of aspirin intake in the Medisafe^©^ app. Moreover, the BMQ and ProMAS instruments were used longitudinally at three time points during pregnancy and postpartum. This longitudinal follow-up provided the first insights into individual medication beliefs and adherence trajectories throughout the perinatal period. To evaluate the evolution in women’s beliefs about medicines during pregnancy, the pregnancy-specific statements of the BMQ were also administered in the postpartum phase, instead of the lactation-specific statements. The self-reported adherence rates of aspirin intake were not checked by the researchers during the study period, and the lack of intermediate intervention or interference can neither have indirectly nor unintentionally increased women’s medication adherence, further enhancing the objectivity of the results. Finally, the Medisafe app has a high ranking in terms of engagement, functionality, desirable characteristics, and overall quality (Carmody et al., 2019; González de León et al., 2021). The app is readily available to the public and can be used outside of study contexts. Study participants indicated that using the app was easy, straightforward, and user-friendly.

This study also has some limitations. A first limitation concerns selection bias and the lack of external validity of the findings. The study cohort consisted of a rather limited sample of women at high-risk of developing GHD who were part of a clinical trial and, hence, received additional care and follow-up, which may also be reflected in the favorable pregnancy and neonatal outcomes observed in this cohort. Participants may have been more motivated to use their medication correctly, already before, as well as during their study participation (Hawthorne effect or information bias). They were also well-informed about their risk and consequences of GHD and the beneficial effect of aspirin as a prophylaxis for PE, potentially leading to high (er) adherence rates compared to the real-world setting. This effect may have been further enhanced by the use of the Medisafe app and its notifications, which likely minimized instances of forgetting to take aspirin. Furthermore, the study design did not allow for a control group without the reminder function of the app, which might have increased overall adherence. Second, with regard to the socio-economic background of the participants, no details on education level or occupation (e.g., occupation in healthcare) were available, although both variables have been described as main determinants of medication beliefs (Nordeng et al., 2010; Ceulem et al., 2019; Zaki and Albarraq, 2014). Third, the ProMAS instrument was used for the first time in the pregnant population, and its statements were not adapted for ‘aspirin’ as the medication of interest. Hence, our results are exploratory, should be cautiously interpreted, and require further investigation. Furthermore, a small number of women (17%) completed the ProMAS on the same day aspirin was prescribed. Fourth, only a minority of the participants completed both instruments at all time points, limiting the internal validity of the findings related to the evolution of beliefs and adherence in the perinatal period. Finally, medication intake was not questioned postpartum.

### 4.4 Future perspectives

The study could be replicated with a large(r) sample size of women at risk for developing GHD. The study should preferably be performed in a ‘real-world’ context outside the setting of a clinical trial. Future studies should also focus on the clinical value of the ProMAS instrument by assessing the relationship of the ProMAS scores early in pregnancy with self-reported intake of medication/aspirin in pregnancy, and pregnancy and neonatal outcomes. Since this study only used the MediSafe^©^ app for the first time in the pregnant population, further research is warranted, e.g., to investigate if the use of the app improves medication adherence among (at-risk) pregnant women by providing reminders, offering education, and facilitating communication between patients and healthcare professionals (Morawski et al., 2018; Santo et al., 2017). This is also relevant from a public health and economic perspective as medication reminder apps may have the potential to reduce healthcare costs as a result of improved clinical outcomes (Santo et al., 2017).

### 4.5 Conclusion

Women at risk for GHD have positive beliefs about medication use in general and during pregnancy throughout the perinatal period. While the women’s overall medication beliefs did not seem to be influenced by their pregnancy, a higher awareness of the beneficial effects of medicine use in pregnancy was observed in this specific population. Medication adherence varied across individuals, however, almost maximum adherence rates for aspirin intake during pregnancy were observed, alongside a trend towards decreased adherence in the postpartum period. ProMAS adherence scores during pregnancy and early postpartum positively correlated with self-reported aspirin intake during pregnancy, while higher ProMAS scores were found among women with uncomplicated pregnancies compared to those with complications. These findings are exploratory and underscore the need for further research to evaluate the potential, clinical utility of the ProMAS instrument as a screening tool in early pregnancy to assess medication adherence and identify risks for adverse pregnancy and neonatal outcomes.

## Data Availability

The raw data supporting the conclusions of this article will be made available by the authors, without undue reservation.
