# Supplementary material for: Medication beliefs and adherence during and after pregnancy among women at risk for gestational hypertensive disorders
Source: Front Drug Saf Regul. 2025 Aug 11;5:1610273. doi: 10.3389/fdsfr.2025.1610273 (PMC12443123; doi:10.3389/fdsfr.2025.1610273)
Supplement: Supplementary file 1 [file Table1.docx]

Supplementary Material

# Supplementary Tables

| **Total pregnancies** | | **N = 54** |
| --- | --- | --- |
| Medication | | |
|  | *Doxylamine + pyridoxine* | 13 (24.1%) |
|  | L-thyroxin | 4 (7.4%) |
|  | Penicillin | 3 (5.6%) |
|  | Enoxaparin | 2 (3.7%) |
|  | Insulin | 2 (3.7%) |
|  | Salbutamol | 2 (3.7%) |
|  | Fluticason + vilanterol | 2 (3.7%) |
|  | Omeprazol | 2 (3.7%) |
|  | Propylthiouracil | 1 (1.8%) |
|  | Bisoprolol | 1 (1.8%) |
|  | Fluoxetin | 1 (1.8%) |
|  | Sertralin | 1 (1.8%) |
|  | Quetiapin | 1 (1.8%) |
|  | Azathioprin | 1 (1.8%) |
|  | Prednisolon | 1 (1.8%) |
| Vitamins/nutritional supplement | | |
|  | Multivitamins (+ folic acid) | 26 (48.1%) |
|  | Folic Acid | 11 (20.4%) |
|  | Vitamin D | 5 (9.3%) |
|  | Iron | 3 (5.6%) |
|  | Cranberry-based supplement | 2 (3.7%) |
|  | Multivitamins (without folic acid) | 1 (1.8%) |
|  | Zinc | 1 (1.8%) |
|  | Pro-biotics | 1 (1.8%) |
|  | Barinutrics | 1 (1.8%) |
|  | Homeopatic cough syrup | 1 (1.8%) |
| Data are presented as numbers (%). | | |

Supplementary Table 1: Concomitant medication and/or supplemental intake, apart from low-dose aspirin, of the women who completed the BMQ and/or ProMAS during pregnancy at the time of completion.

Supplementary Table 2: Beliefs about medicines – general statements reported 10-21 days postpartum (N=41).

|  | **(totally) agree** | **uncertain** | **(totally) disagree** |
| --- | --- | --- | --- |
| G1. Doctors use too many medicines. | 8 (19.5%) | 11 (26.8%) | 22 (53.7%) |
| G2. People who take medicines should stop their treatment for a while every now and again. | 7 (17.1%) | 16 (39.0%) | 18 (43.9%) |
| G3. Most medicines are addictive. | 4 (9.8%) | 12 (29.3%) | 25 (61.0%) |
| G4. Natural remedies are safer than medicines. | 8 (19.5%) | 13 (31.7%) | 20 (48.8%) |
| G5. Medicines do more harm than good. | 3 (7.3%) | 4 (9.8%) | 34 (82.9%) |
| G6. All medicines are poisons. | 2 (4.9%) | 3 (7.3%) | 36 (87.8%) |
| G7. Doctors place too much trust on medicines. | 12 (29.3%) | 9 (22.0%) | 20 (48.8%) |
| G8. If doctors had more time with patients they would prescribe fewer medicines. | 12 (29.3%) | 12 (29.3%) | 17 (41.5%) |

Supplementary Table 3: Beliefs about medicines – general statements reported 4-6 months postpartum (N=33).

|  | **(totally) agree** | **uncertain** | **(totally) disagree** |
| --- | --- | --- | --- |
| G1. Doctors use too many medicines. | 6 (18.2%) | 10 (30.3%) | 17 (51.5%) |
| G2. People who take medicines should stop their treatment for a while every now and again. | 4 (12.1%) | 14 (42.4%) | 15 (45.5%) |
| G3. Most medicines are addictive. | 6 (18.2%) | 4 (12.1%) | 23 (69.7%) |
| G4. Natural remedies are safer than medicines. | 6 (18.2%) | 6 (18.2%) | 21 (63.6%) |
| G5. Medicines do more harm than good. | 3 (9.1%) | 2 (6.1%) | 28 (84.8%) |
| G6. All medicines are poisons. | 4 (12.1%) | 2 (6.1%) | 27 (81.8%) |
| G7. Doctors place too much trust on medicines. | 8 (24.2%) | 8 (24.2%) | 17 (51.5%) |
| G8. If doctors had more time with patients they would prescribe fewer medicines. | 10 (30.3%) | 7 (21.2%) | 16 (48.5%) |

Supplementary Table 4: Results of the BMQ general statements completed during pregnancy and postpartum.

|  | **During pregnancy (N = 63)** | **10-21 days postpartum  (N = 41)** | **4-6 months postpartum (N = 33)** |
| --- | --- | --- | --- |
| BMQ general - score overuse (/20) | 10.6 ± 2.9 | 10.6 ± 3.5 | 10.5 ± 3.9 |
| BMQ general - score harm (/20) | 8.8 ± 2.2 | 8.7 ± 2.4 | 8.8 ± 2.8 |
| Continuous data are given as mean ± standard deviation or median (interquartile range), depending on the normality. Categorical data are presented as numbers (%). BMQ: Beliefs about Medicine Questionnaire | | | |

Supplementary Table 5: Beliefs about medicines – pregnancy-specific statements reported 10-21 days postpartum (N=39).

|  | **(totally) agree** | **uncertain** | **(totally) disagree** |
| --- | --- | --- | --- |
| P1. All medicines can be harmful for the fetus. | 4 (10.3%) | 8 (20.5%) | 27 (69.2%) |
| P2. Even if I am ill and if not pregnant would have taken a medicine, I believe it’s better for the fetus that I refrain from using medicines during pregnancy. | 19 (48.7%) | 4 (10.3%) | 16 (41.0%) |
| P3. I have a higher threshold for using medicines when I am pregnant than when I am not pregnant. | 34 (87.2%) | 1 (2.6%) | 4 (10.3%) |
| P4. Thanks to treatment with medicines during pregnancy, lives of many unborn children are saved each year. | 16 (41.0%) | 22 (56.4%) | 1 (2.6%) |
| P5. It is better for the fetus that I use medicines and get well than to have an untreated illness during pregnancy. | 21 (53.8%) | 11 (28.2%) | 7 (17.9%) |
| P6. Doctors prescribe too many medicines to pregnant women. | 2 (5.1%) | 10 (25.6%) | 27 (69.2%) |
| P7. Natural remedies can generally be used by pregnant women. | 10 (25.6%) | 22 (56.4%) | 7 (17.9%) |
| P8. Pregnant women should preferably use natural remedies during pregnancy. | 11 (28.2%) | 16 (41.0%) | 12 (30.8%) |
| P9. Pregnant women should not use natural remedies without the consent of a doctor. | 28 (71.8%) | 8 (20.5%) | 3 (7.7%) |

Supplementary Table 6: Beliefs about medicines – pregnancy-specific statements reported 4-6 months postpartum (N=34).

|  | **(totally) agree** | **uncertain** | **(totally) disagree** |
| --- | --- | --- | --- |
| P1. All medicines can be harmful for the fetus. | 4 (11.8%) | 1 (2.9%) | 29 (85.3%) |
| P2. Even if I am ill and if not pregnant would have taken a medicine, I believe it’s better for the fetus that I refrain from using medicines during pregnancy. | 13 (38.2%) | 3 (8.8%) | 18 (52.9%) |
| P3. I have a higher threshold for using medicines when I am pregnant than when I am not pregnant. | 30 (88.2%) | 0 (0.0%) | 4 (11.8%) |
| P4. Thanks to treatment with medicines during pregnancy, lives of many unborn children are saved each year. | 22 (64.7%) | 11 (32.4%) | 1 (2.9%) |
| P5. It is better for the fetus that I use medicines and get well than to have an untreated illness during pregnancy. | 24 (70.6%) | 7 (20.6%) | 3 (8.8%) |
| P6. Doctors prescribe too many medicines to pregnant women. | 4 (11.8%) | 7 (20.6%) | 23 (67.6%) |
| P7. Natural remedies can generally be used by pregnant women. | 15 (44.1%) | 10 (29.4%) | 9 (26.5%) |
| P8. Pregnant women should preferably use natural remedies during pregnancy. | 13 (38.2%) | 12 (35.3%) | 9 (26.5%) |
| P9. Pregnant women should not use natural remedies without the consent of a doctor. | 23 (67.6%) | 6 (17.6%) | 5 (14.7%) |

Supplementary Table 7: Statistical output of the Spearman’s Rho correlation analysis of the ProMAS adherence sum scores during pregnancy and the “minimal” self-reported aspirin intake (N=55).

| **Correlations** | | | | |
| --- | --- | --- | --- | --- |
|  | | | MIN_COMPLIANCE | PROMAS_BL_SUM |
| Spearman's rho | MIN_COMPLIANCE | Correlation Coefficient | 1,000 | ,524^**^ |
|  |  | Sig. (2-tailed) | . | <,001 |
|  |  | N | 67 | 55 |
|  | PROMAS_BL_SUM | Correlation Coefficient | ,524^**^ | 1,000 |
|  |  | Sig. (2-tailed) | <,001 | . |
|  |  | N | 55 | 61 |
| **. Correlation is significant at the 0.01 level (2-tailed). | | | | |

Supplementary Table 8: Statistical output of the Spearman’s Rho correlation analysis of the ProMAS adherence sum scores during pregnancy and the “maximal” self-reported aspirin intake (N=55).

| **Correlations** | | | | |
| --- | --- | --- | --- | --- |
|  | | | PROMAS_BL_SUM | MAX_COMPLIANCE |
| Spearman's rho | PROMAS_BL_SUM | Correlation Coefficient | 1,000 | ,297^*^ |
|  |  | Sig. (2-tailed) | . | ,028 |
|  |  | N | 61 | 55 |
|  | MAX_COMPLIANCE | Correlation Coefficient | ,297^*^ | 1,000 |
|  |  | Sig. (2-tailed) | ,028 | . |
|  |  | N | 55 | 67 |
| *. Correlation is significant at the 0.05 level (2-tailed). | | | | |

Supplementary Table 9: Statistical output of the Spearman’s Rho correlation analysis of the ProMAS adherence sum scores at 10-21 days postpartum and the “minimal” self-reported aspirin intake (N=35).

| **Correlations** | | | | |
| --- | --- | --- | --- | --- |
|  | | | MIN_COMPLIANCE | PROMAS_PP1_SUM |
| Spearman's rho | MIN_COMPLIANCE | Correlation Coefficient | 1,000 | ,761^**^ |
|  |  | Sig. (2-tailed) | . | <,001 |
|  |  | N | 35 | 35 |
|  | PROMAS_PP1_SUM | Correlation Coefficient | ,761^**^ | 1,000 |
|  |  | Sig. (2-tailed) | <,001 | . |
|  |  | N | 35 | 38 |
| **. Correlation is significant at the 0.01 level (2-tailed). | | | | |

Supplementary Table 10: Statistical output of the Spearman’s Rho correlation analysis of the ProMAS adherence sum scores at 10-21 days postpartum and the “maximal” self-reported aspirin intake (N=35).

| **Correlations** | | | | |
| --- | --- | --- | --- | --- |
|  | | | PROMAS_PP1_SUM | MAX_COMPLIANCE |
| Spearman's rho | PROMAS_PP1_SUM | Correlation Coefficient | 1,000 | ,432^**^ |
|  |  | Sig. (2-tailed) | . | ,010 |
|  |  | N | 38 | 35 |
|  | MAX_COMPLIANCE | Correlation Coefficient | ,432^**^ | 1,000 |
|  |  | Sig. (2-tailed) | ,010 | . |
|  |  | N | 35 | 35 |
| **. Correlation is significant at the 0.01 level (2-tailed). | | | | |

|  |  | Uncomplicated pregnancy outcome (N = 27) | Pregnancy complicated by GHD and/or PTB and/or SGA (N = 24)* | P-value |
| --- | --- | --- | --- | --- |
| ProMAS pregnancy adherence rate | Medium-low (0-9) | 10 (37.0%) | 15 (62.5%) | 0.069 |
|  | Medium-high  (10-18) | 17 (63.0%) | 9 (37.5%) |  |
|  | Sum score | 10.9 ± 3.5 | 8.9 ± 4.1 | 0.065 |
| Minimal self-reported aspirin intake | ≥ 80% | 20 (74.1%) | 19 (79.2%) | 0.669 |
|  | ≥ 90% | 14 (51.9%) | 14 (58.3%) | 0.642 |
|  | Level | 90.5 (79.4 – 97.5) | 92.9 (80.3 – 96.6) | 0.784 |
| Maximal self-reported aspirin intake | ≥ 80% | 27 (100.0%) | 23 (95.8%) | 0.471 |
|  | ≥ 90% | 27 (100.0%) | 23 (95.8%) | 0.471 |
|  | Level | 100 (99.1 – 100) | 100 (99.1 – 100) | 0.853 |
| *ProMAS: Probabilistic Medication Adherence Scale; GHD: gestational hypertensive disorders; PTB: preterm birth; SGA: small for gestational age neonate. * In case the subject was diagnosed with any subtype of GHD in combination with PTB, and/or SGA, the subject was categorized under ‘GHD’. This was the case for four subjects: two women also delivered preterm, and two other women also had an SGA neonate. None of the subjects classified under ‘SGA’ or ‘PTB’ had any other complication. Continuous data are given as mean ± standard deviation or median (interquartile range), depending on the normality. Categorical data are presented as numbers (%).* *P-value for continuous data was assessed via one-sided Independent Samples T-test or the Mann-Whitney U test, depending on the normality. P-value for binary data was assessed via the Chi-squared test. A p-value <0.05 was considered statistically significant.* | | | | |

Supplementary Table 11: Comparison of the ProMAS sum scores during pregnancy and the self-reported aspirin intake according to the pregnancy outcome (N=51).

Supplementary Table 12: Overview of the ProMAS sum scores at 10-21 days postpartum and the self-reported aspirin intake according to the pregnancy outcome (N=34).

|  | ProMAS early-postpartum adherence rate | | Minimal self-reported aspirin intake | | Maximal self-reported aspirin intake | |
| --- | --- | --- | --- | --- | --- | --- |
|  | Medium-low (0-9) | Medium-high  (10-18) | ≥ 80% | ≥ 90% | ≥ 80% | ≥ 90% |
| Uncomplicated term pregnancy outcome (N = 20) | 10 (50.0%) | 10 (50.0%) | 17 (85.0%) | 13 (65.0%) | 20 (100.0%) | 20 (100.0%) |
| Pregnancy complicated by GHD or PTB (N = 14) | 7 (50.0%) | 7 (50.0%) | 11 (78.6%) | 8 (57.1%) | 13 (92.8%) | 12 (85.7%) |
| *GHD (N = 12)* | 5 (41.7%) | 7 (58.3%) | 10 (83.3%) | 8 (66.7%) | 12 (100.0%) | 11 (91.7%) |
| *PTB (N = 2)* | 2 (100.0%) | 0 (0.00%) | 1 (50.0%) | 0 (0.00%) | 1 (50.0%) | 1 (50.0%) |
| *ProMAS: Probabilistic Medication Adherence Scale; GHD: gestational hypertensive disorders; PTB: preterm birth.* | | | | | | |

Supplementary Table 13: Comparison of the ProMAS sum scores assessed at 10-21 days postpartum and the self-reported aspirin intake according to the pregnancy outcome (N=34).

|  |  | Uncomplicated pregnancy outcome (N = 20) | Pregnancy complicated by GHD or PTB (N = 14) | P-value |
| --- | --- | --- | --- | --- |
| ProMAS pregnancy adherence rate | Medium-low (0-9) | 10 (50.0%) | 7 (50.0%) | 1.000 |
|  | Medium-high  (10-18) | 10 (50.0%) | 7 (50.0%) |  |
|  | Sum score | 9.5 ± 4.0 | 9.6 ± 4.8 | 0.926 |
| Minimal self-reported aspirin intake | ≥ 80% | 17 (85.0%) | 11 (78.6%) | 0.672 |
|  | ≥ 90% | 13 (65.0%) | 8 (57.1%) | 0.643 |
|  | Level | 94.9 (87.2 – 98.8) | 94.6 (80.5 – 99.5) | 0.752 |
| Maximal self-reported aspirin intake | ≥ 80% | 20 (100.0%) | 13 (92.8%) | 0.412 |
|  | ≥ 90% | 20 (100.0%) | 12 (85.7%) | 0.162 |
|  | Level | 100 (99.3 – 100) | 99.7 (94.0 – 100) | 0.119 |
| *ProMAS: Probabilistic Medication Adherence Scale; GHD: gestational hypertensive disorders; PTB: preterm birth. Continuous data are given as mean ± standard deviation or median (interquartile range), depending on the normality. Categorical data are presented as numbers (%).* *P-value for continuous data was assessed via one-sided Independent Samples T-test or the Mann-Whitney U test, depending on the normality. P-value for binary data was assessed via the Chi-squared test. A p-value <0.05 was considered statistically significant.* | | | | |
